# COMPARISON OF ANTHROPOMETRIC MEASUREMENTS OF CHILDREN LIVING IN THE DOMINICAN REPUBLIC AND PERU

**DOI:** 10.51894/001c.123117

**Published:** 2024-08-30

**Authors:** Zack Murphy

**Affiliations:** 1 College of Osteopathic Medicine Michigan State University

## 93

### INTRODUCTION/BACKGROUND

The current standard for pediatric growth is based on the World Health Organization’s (WHO) growth charts. However, Brazil is the only country representing Latin America in this data. Therefore, this study analyzes the accuracy of the WHO pediatric growth charts in the setting of two distinct Latin countries, the Dominican Republic (DR) (a Caribbean population) and Peru (an Andean population).

### OBJECTIVES/HYPOTHESIS

Our first hypothesis is that the data from the WHO is not representative of all Latin American countries, and we propose country specific grow­th charts to accurately represent each population. Our second hypothesis (based on previous results collected in 2022), is that children in the DR will have increased measurements for both weight and height compared to those in Peru.

### METHODS

Cross-sectional measurements were collected among 107 children in the DR and 119 children in Peru, aged two months to five years old in 2022 and 2023. Weight and height/length were taken following WHO guidelines, using an aluminum scale mount, digital baby scale, and measurement tape.

### RESULTS

Comparison of mean differences using independent samples t-test of DR and Peru data revealed a significant difference in z-scores for weight and height between the two populations. Bland-Altman analysis of DR children’s weight shows an overall bias of 0.29kg heavier, while height shows an overall bias of being 1.51 cm taller. Bland-Altman analysis of Peru children’s weight shows an overall bias of 0.51 kg lighter, while height shows an overall bias of being 3.39 cm shorter. Considering expected weights for given heights, analysis showed no bias.

### DISCUSSION/CONCLUSION

The t-test results show significant differences between the DR and Peru. On average DR children are heavier and taller while Peru children are lighter and shorter which supports our second hypothesis. This indicates the possible need for additional WHO growth charts that are representative of different geographic locations and cultures. Although the comparison with the WHO growth charts show some bias for weight and height in both countries, the charts still capture the normal range of health in most cases. Thus, they cannot be rejected at this time.

